# Metal Binding to Tau Protein: Physiological and Pathological Relevance

**DOI:** 10.1021/acs.biochem.6c00323

**Published:** 2026-07-11

**Authors:** Liliana Quintanar, Gerardo U. Juárez-Romero, Gala R. López-Herrera

**Affiliations:** † Center for Research in Aging, Center for Research and Advanced Studies (Cinvestav), 14330 Mexico City, Mexico; ‡ Department of Chemistry, Cinvestav, 07360 Mexico City, Mexico

## Abstract

The microtubule-associated protein tau is an intrinsically disordered protein that exhibits a remarkable diversity of functions, while its amyloid aggregation is the hallmark of tauopathies, including Alzheimer′s disease. This perspective article discusses recent advances in understanding copper and zinc binding to tau, the nature of the metal binding sites, and its physiological and pathological relevance. First, an argument for a potential role of metal binding in the interactome and diverse functionality of tau is provided, since metal coordination occurs in regions of tau engaged in its interaction with key binding partners. Then, the role of metal binding to tau in modulating its structural dynamics and amyloid aggregation behavior is discussed, with an emphasis on a potential role of metal ions in the morphological diversity of tau fibrils. Finally, the interplay between metal binding and post-translational modifications (PTMs) of tau is examined in physiological and pathological contexts, discussing how PTMs may influence metal coordination and how metals may modulate PTM processing of tau. Overall, this perspective delineates the exciting future of bioinorganic research related to tau protein, underscoring the importance of investigating the role of metal ions in tau biology as functional modulators and/or drivers of pathological transitions, linking metal homeostasis to tau physiology and disease.

## Graphical abstract

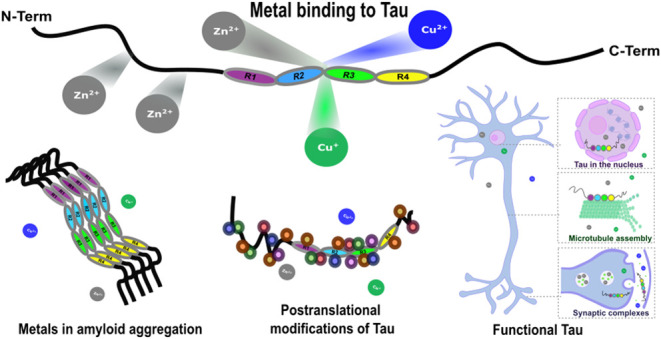

## 1 Introduction

Proteinopathies are a group of diseases triggered by the aggregation of proteins that normally fulfill essential physiological functions.^1^ These proteins become pathologically active upon structural changes that promote misfolding, self-association, and accumulation within tissues.^2^ In the central nervous system, such aberrant aggregation is neurotoxic and leads to progressive neurodegeneration. Indeed, neurodegenerative disorders such as prion disease, Parkinson’s and Alzheimer’s diseases, are proteinopathies associated with the aggregation of the prion protein, α-synuclein, amyloid-β and tau, respectively.^1^ All these proteins are also metal binding proteins, while metal ions have been linked to the associated proteinopathies, underscoring the importance of studying the interplay between metal binding, protein folding, and aggregation.

The homeostasis of essential metals, such as iron, copper, and zinc, is altered in Alzheimer’s disease (AD) and other tauopathies, and hence, these metals are thought to play an important pathological role in these diseases.^3,4^ In AD, total copper levels are decreased in brain tissue, while increasing blood serum.^5^ Zinc levels, on the other hand, are elevated in the AD brain, particularly in the later stages of the disease.^6^ Iron levels are also elevated in the AD brain, with accumulation in the hippocampus and cerebral cortex.^7^ Moreover, metal ions are known to cause synaptic disruption, oxidative stress and are associated with amyloid aggregation.^8^

Early studies revealed that extracellular amyloid plaques from AD brains, composed mostly of amyloid-β peptide (Aβ), accumulate transition metals such as iron, copper and zinc.^9^ Similarly, there are a couple of studies that reveal the presence of copper and zinc in the neurofibrillary tangles (NFTs) of patients with AD, linking these metal ions with tau aggregates.^10,11^ These findings suggest that AD-related Aβ peptide and tau protein may be metal binding proteins. Indeed, over the past two decades, extensive research has focused on the metal-binding properties of the Aβ peptide, which can bind copper and zinc with high affinity, and the structural details of the resulting metal-Aβ have been elucidated.^4,12^ Metal binding to Aβ impacts its amyloid aggregation and toxicity.^13^ In addition, copper-Aβ species exhibit redox activity that can contribute to the generation of reactive oxygen species (ROS) and/or participate in metal-catalyzed oxidation of the Aβ peptide or other relevant substrates, exacerbating neuronal damage.^14^ In contrast to the case of Aβ, research on the metal-binding properties of tau protein has been scarce.

In this perspective article, the main findings of a recent spectroscopic study that reveals important structural insights into the copper and zinc binding sites of tau are presented, while the physiological and pathological relevance of these metal-tau interactions are discussed. First, an argument for a potential role of metal binding in tau′s interactome is presented. Then, the role of metal binding in the amyloid aggregation of tau and the morphological diversity of tau fibrils is discussed. Finally, the interplay between metal binding and post-translational modifications tau is examined in physiological and pathological contexts. Overall, this perspective delineates the exciting future of bioinorganic research related to tau protein.

## 2 Tau Protein and Its Metal Binding Properties

Tau is a protein that belongs to the family of microtubule associated proteins (MAP), described as an essential protein for microtubule assembly.^15^ In the human brain, six main isoforms are generated by alternative splicing of the MAPT gene. These isoforms range from 352 to 441 amino acids and differ by the presence or absence of inserts encoded by exons 2, 3, and 10. Splicing of exons 2 and 3 produces the 0N, 1N, and 2N amino-terminal variants, while inclusion or exclusion of exon 10 generates isoforms with four (4R) or three (3R) C-terminal repeats, respectively (Figure 1).^16^

**Figure 1:**
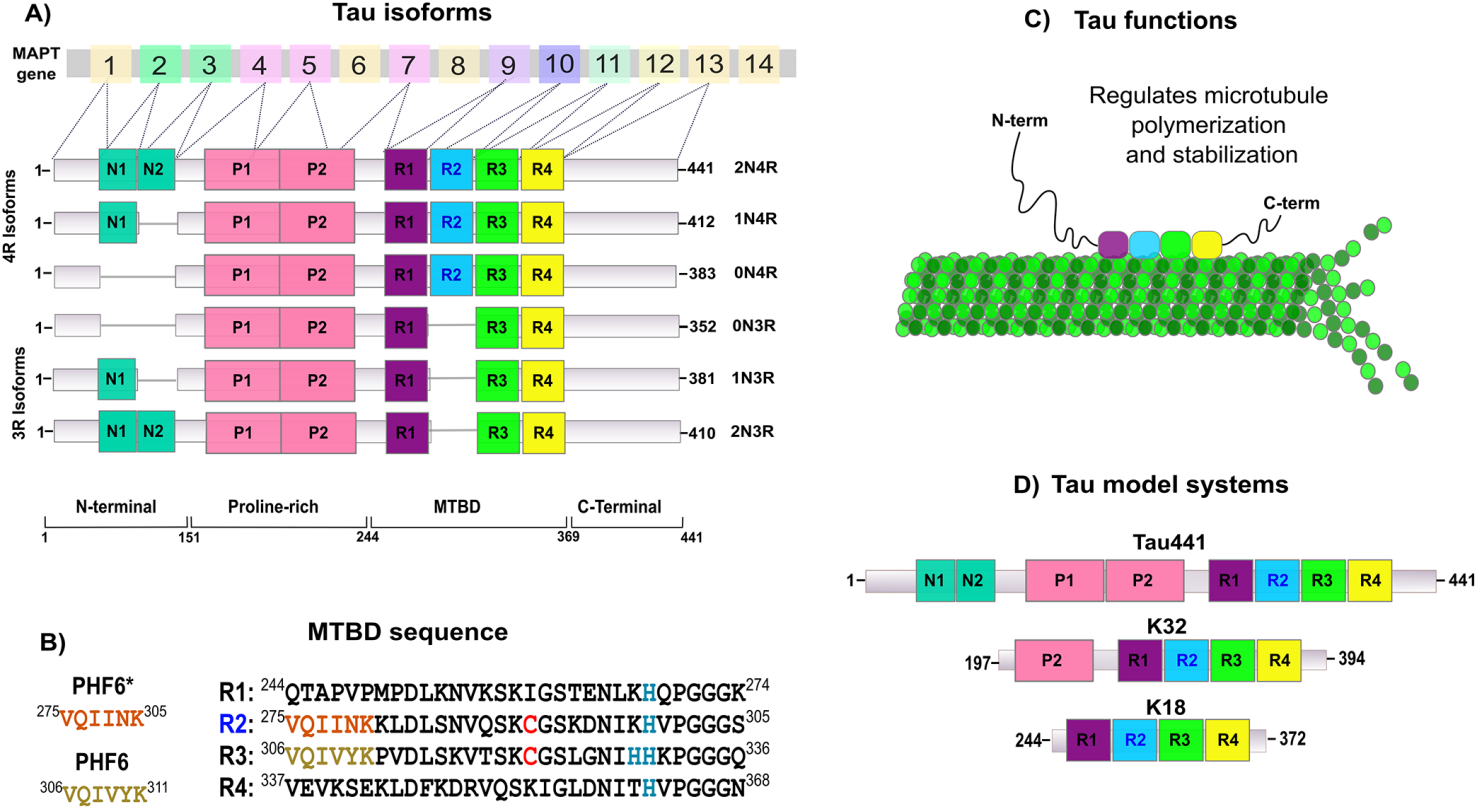
Isoforms, functions and experimental models in the study of tau. (A), Schematic representation of the *MAPT* gene illustrating alternative splicing events that produce tau isoforms with either three (3R) or four (4R) microtubule-binding repeats, determined by the exclusion or inclusion of the second repeat (R2) encoding by exon 10. (B), Amino acid sequence of the Microtubule Binding Domain (MTBD) showing the repeat regions and the location of the aggregation-prone PHF motifs (PHF6 and PHF6*), which are critical for tau fibrillization. Histidine and Cysteine residues are shown in blue and red, respectively. (C), Illustration of tau function in promoting and stabilizing microtubule assembly through binding to tubulin polymers within the MTBD. (D), Commonly used experimental constructs for the study of tau structure and aggregation, including full-length tau (Tau441) and truncated fragments encompassing the repeat domains, such as K18 (four-repeat construct) and K32, widely used as minimal models to investigate tau aggregation and filament formation.

While several aspects of tau protein have been extensively investigated, including its interaction with microtubules, aggregation behavior, and post-translational modifications, its interaction with metal ions has received comparatively less attention. The discovery that metals, such as copper^10^ and zinc,^11^ are present in NFTs prompted some interest in probing the metal binding properties of tau. Early studies by isothermal titration calorimetry revealed that monomeric full-length tau can bind metal ions, containing a single Cu^2+^ binding site with an affinity in the low micromolar range,^17^ and multiple binding sites for Zn^2+^ ions: one of them with a low micromolar affinity^18,19^ and up to three additional low-affinity sites in the high micromolar range.^18^

Recently, a study provided structural insights into the Cu^2+^, Cu^+^, and Zn^2+^ binding sites of the full-length tau protein (Tau441), using different spectroscopic techniques.^20^ Tau441 contains a single high-affinity Cu^2+^ site within the microtubule-binding domain (MTBD), defined by a bis-histidine motif (His329/His330) in R3 together with His299 in R2 (Figure 2A). Upon reduction, this site rearranges into a Cu^+^ center with trigonal coordination involving His299, Cys322, and an additional ligand, likely Cys291 or His329/330 (Figure 2B). Zinc binding sites involve different domains, a high-affinity MTBD site that overlaps the copper-binding region and two additional lower-affinity sites in the N-terminal domain. The Zn^2+^ high-affinity site in the MTBD region involves the bis-His motif, His299, and Cys322 (Figure 2C), while the two N-terminal sites involve LxxxL motifs, where L represents amenable ligands for Zn^2+^, such as His, Asp, or Glu residues. Structural models for the copper and zinc binding sites at the MTBD region were built using Alphafold3.0,^21^ these models are consistent with spectroscopically derived information and show the possible conformation changes in the local structure of the protein upon metal coordination. Structural models suggest the formation of an α-helix, where Lys residues in R2 and R3 domains, that are key in microtubule interactions and amyloid formation,^22^ might be aligned along one side of the helix (Figure 2). In addition, the aggregation-prone PHF6 motif could be positioned within a loop region located adjacent to the metal coordination sites of tau. If these structural models were correct, metal binding could impact protein-microtubule interactions and/or amyloid aggregation.

**Figure 2:**
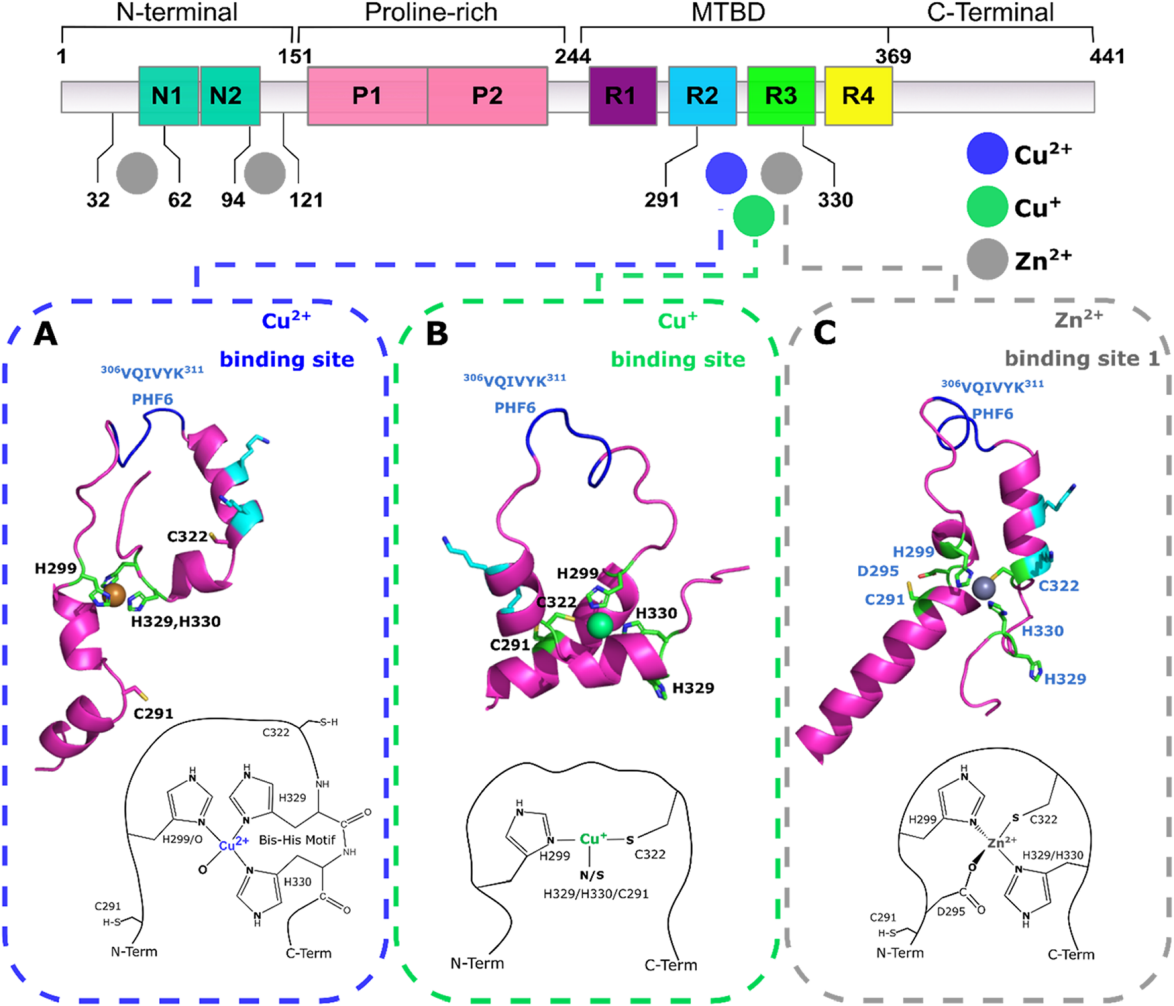
Metal Binding properties of Tau441. Cu^2+^, Cu^+^ and Zn^2+^ coordination sites in Tau441 are identified by blue, green and gray circles, respectively. Coordination modes (derived from spectroscopic results) and structural models generated by Alphafold3.0 for the Cu^2+^ (A), Cu^+^ (B) and Zn^2+^ (site 1) (C) binding sites in Tau441 are represented. For the Alphafold3.0 models, only the R2 and R3 repeats were used, putative metal binding residues are shown in green, the PHF6 sequence is shown in dark blue, and Lys residues that interact with heparin are in cyan. Figure adapted from ref 20 with permission of Royal Society of Chemistry.

The convergence of copper and zinc coordination within the MTBD suggests a potential physiological role for metals in modulating tau–tubulin interactions and microtubule dynamics. Given the intrinsically disordered nature of Tau441, metal coordination may serve as a structural modulator, stabilizing specific conformational states that facilitate protein–protein or protein–nucleic acid interactions with physiological or pathological relevance, as discussed below.

## 3 Metal Binding to Tau and Its Interactome

Research over the past two decades has expanded the functional repertoire of tau far beyond microtubule regulation. Tau is now recognized as a multifunctional protein that participates in cytoskeletal dynamics, axonal transport, synaptic signaling, nuclear maintenance, RNA metabolism, and proteostasis.^23^ These diverse roles are mediated through a complex and dynamic interactome that varies with cellular context,^24^ post-translational modifications,^25^ isoform expression,^26^ and pathological mutations.^24^ Tau interactomes have been characterized using complementary approaches such as coimmunoprecipitation.^26–28^ Some of these proteins are metal-binding proteins and are shown in Figure 3, classified by the region of tau where they interact. In this section, the possible role of metal binding in the interactome of tau and its relevance in different cellular compartments will be discussed.

**Figure 3:**
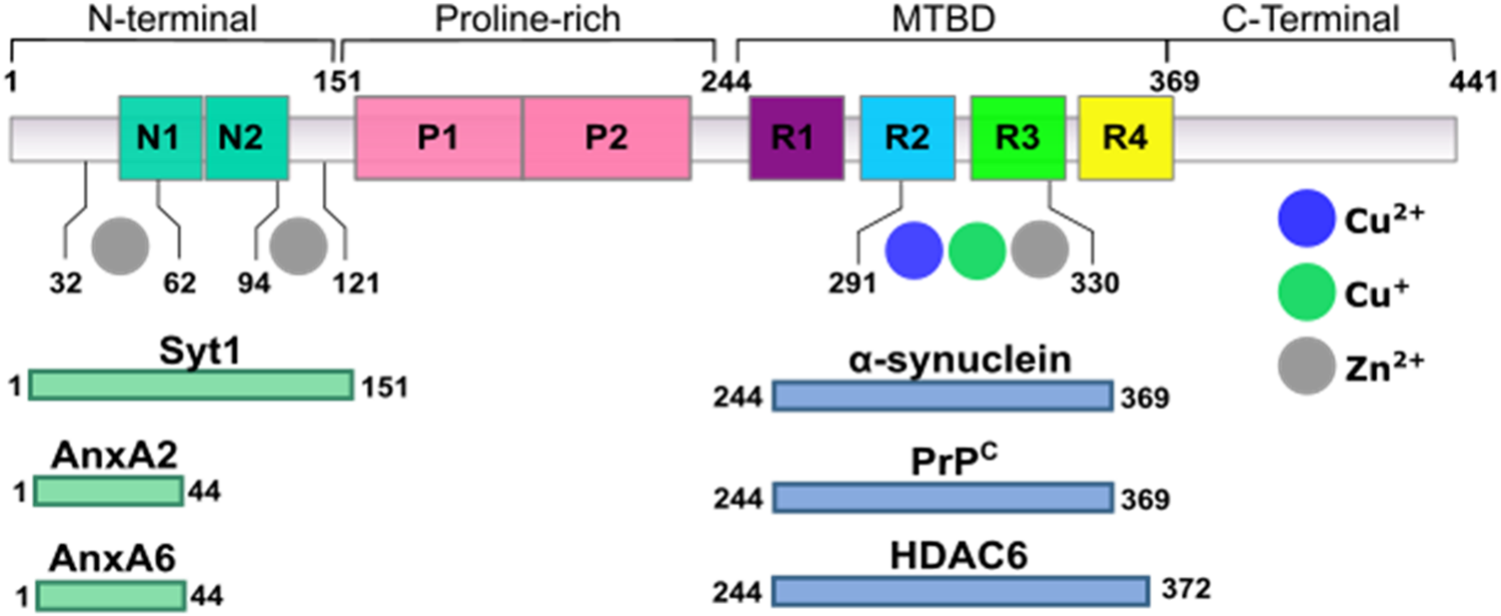
The interactome of tau. This figure illustrates representative protein partners known to associate with tau across distinct domains including synaptotagmin (Syt1), annexin A2 (Anxa2), annexin A6 (Anxa6), α-synuclein, cellular prion protein (PrP^C^) and human histone deacetylase 6 (HDAC6). These proteins are known to bind metal ions such as Ca^2+^, Zn^2+^ or Cu^2+^, indicating potential mechanistic links between tau protein–protein interactions and cellular metal homeostasis.

### 3.1 Nuclear Interactome of Tau

Tau is also present in the nucleus, where it can associate with DNA, histones, RNA, and other nuclear proteins, suggesting roles in genome protection, chromatin organization, and the regulation of gene expression.^23,29,30^ The MTBD, particularly the R1–R4 repeats, mediates tau–DNA interactions through electrostatic contacts between the positively charged Lys and Arg residues in MTBD and the negatively charged DNA backbone.^29,31^ Recent work has further demonstrated that tau can bind DNA with high affinity at nanomolar concentrations and promote the formation of dynamic tau–DNA co-condensates,^32^ which may serve as platforms that organize microtubules and facilitate their interaction with chromosomes. These condensates localize near centromeres during mitosis and may contribute to the early engagement of mitotic spindles with chromosomes. Additionally, tau phosphorylation, an important post-translational modification of the protein, modulates these interactions and can impair chromosome alignment during cell division.

The nucleus is a zinc-rich cellular compartment; it is estimated to contain between 30 and 40% of total intracellular zinc, although most of it would be bound to transcriptional factors and other proteins.^33^ Hence, the MTBD Zn^2+^-binding site of tau (Figure 2C) may become occupied under physiological conditions, potentially influencing tau–DNA interactions. Indeed, zinc binding has been shown to alter the mode of tau association with DNA, shifting from minor-groove to major-groove binding, which likely reflects a metal-induced conformational change in the protein.^34^ Understanding the structural consequences of zinc binding to Tau441 would require extensive structural studies; however, structural predictions from AlphaFold3 models of the Zn^2+^-bound protein suggest that Lys residues within the R3 repeat may align along one face of an α-helix.^20^ Such an arrangement could enhance electrostatic interactions with DNA, facilitating the formation of tau–DNA assemblies within the nucleus.

Proximity labeling techniques, such as BioID and APEX, have shown that tau interacts in the nucleus with RNA-binding proteins, such as RNA-binding protein FUS and heterogeneous nuclear ribonucleoproteins (hnRNPs).^24,35^ This observation suggests a role of tau in RNA metabolism, stress granule dynamics, and translational control.^36^ Tau interacts extensively with molecular chaperones, as well as components of the ubiquitin–proteasome system.^35^ The impact of metal binding to tau in these protein–protein interactions has not been studied, even though they regulate tau folding, degradation, and aggregation propensity.^27^

### 3.2 Cytosolic Interactome of Tau

Tau interacts with several cytosolic proteins involved in cytoskeletal regulation and cellular proteostasis.^35^ One well-characterized example is the histone deacetylase HDAC6, a cytosolic Zn^2+^-dependent deacetylase that regulates microtubule dynamics and protein quality control by deacetylating α-tubulin and promoting the aggresome–autophagy clearance of misfolded proteins. The tau-HDAC6 interaction does not involve the zinc active site of HDAC6 domain (named BUZ); consistently, tau is known to be a substrate for HDAC6, which deacetylates Lys residues at the MTBD domain, specifically Lys311 and Lys321.^37^ Here, zinc binding to tau at the MTBD site (Figure 2C) may facilitate its interaction with HDAC6 by its effect in the local conformation of the region containing these Lys residues. Interestingly, HDAC6 expression is significantly increased in AD brain,^37^ and has become an important therapeutic target. HDAC6 inhibitors favor clearance of amyloid plaques and cause a decrease in tau aggregation.^38^ Most of these inhibitors are hydroxamates that bind at the zinc BUZ active site of HDAC6, blocking deacetylase activity; however, hydroxamates display very high affinity for Zn^2+^ ions (between 0.1 to 100 nM) and they would also take away the zinc at the MTBD site, possibly interfering with the HDAC6-tau interaction.^39^ The latter could be an overlooked mechanism of action for these HDAC6 inhibitors with therapeutic potential in AD.

### 3.3 Tau Interactome with Synaptic Machinery Proteins

Neuronal activity has been shown to trigger release of tau into the extracellular space.^40^ Activity-dependent tau release is inhibited by tetanus toxin, which disrupts SNARE complex function, supporting a role for presynaptic vesicle fusion machinery in tau release.^40^ Consistent with this mechanism, neuronal depolarization induces a local influx of Ca^2+^ at presynaptic terminals, which activates synaptotagmin-1 and promotes its interaction with the SNARE proteins syntaxin-1 and SNAP-25, to drive vesicle fusion.^41^ Neuronal activity also enhances the interaction of tau with SNARE and synaptic vesicle proteins, suggesting that direct protein–protein interactions with the vesicle fusion machinery may regulate activity-dependent tau release.^24^

Indeed, tau has been reported to interact with several synaptic vesicle proteins, including Synaptotagmin-1 (Syt1) with evidence pointing to the N-terminal region of tau as one of the interaction interfaces.^42^ Syt1 is a Ca^2+^-binding protein that functions as the principal calcium sensor for fast synaptic vesicle exocytosis.^43^ The interaction between tau and Syt1 is suggested to occur in proximity to the Ca^2+^-binding domains, as indicated by APEX-based ubiquitination mapping with the N-terminus of tau.^24,42^ This raises the question of a possible competition between tau and calcium for the site. Regardless, although tau release appears largely nonexosomal, synaptic vesicle fusion may transiently alter the composition of the presynaptic membrane and bring cytosolic tau closer to the plasma membrane, facilitating its unconventional secretion.

In this sense, it is important to mention that zinc concentrations vary across subcellular compartments and are tightly regulated to maintain cellular function. Although the total zinc concentration in cells is relatively high, in the range of 200–300 μM, most zinc is bound to proteins or stored in organelles.^44^ The cytosol contains very low levels of free zinc, typically in the picomolar to nanomolar range, due to a strong buffering effect by zinc-binding proteins.^45^ In neuronal cells, a subset of glutamatergic neurons is known to corelease zinc together with glutamate during synaptic transmission. These neurons contain specialized zinc-containing vesicles that accumulate high concentrations of the metal, using the zinc transporter ZnT3.^46^ However, the exact concentration of zinc inside the vesicle lumen has not been directly measured; current estimates suggest millimolar intravesicular concentrations of at least 1 mM.^47^ It is plausible that, under these conditions, tau release into the synaptic cleft would be in its zinc-bound form.

Additional proteins present in the presynaptic compartment that interact with tau include members of the annexin family (Figure 3), which are a family of calcium-dependent membrane-binding proteins implicated in membrane lipids organization, exocytosis and endocytosis.^48^ Anexins need to be in their Ca-bound form to interact with tau. While it is not clear if the site of interaction of these proteins with tau coincides or not with their Ca binding motifs, it is well-known that Ca plays an important structural role in many proteins, and in the case of anexins Anx2 and Anx6, it is proposed that Ca binding induces a conformation that favors their interaction with tau.^49^ On the other hand, given that these proteins interact with tau at the N1/N2 region, where at least two zinc binding sites are located, it would be interesting to test if zinc binding to tau would impact its interaction with these Ca binding proteins. Calcium and zinc levels change during vesicle release in the presynaptic terminal; hence, tau–annexin complexes may be dynamically regulated by metal ion binding during synaptic transmission.

### 3.4 Pathological Interactome of Tau

Finally, many aggregation-prone proteins interact with tau at is MTBD, including α-synuclein (AS), the main proteic component of Lewi bodies in Parkinson′s disease. AS is a metal binding protein and it is important for synaptic vesicle function.^50^ AS specifically binds copper ions with high affinity at its N-terminal region, while many divalent metal ions bind with lower affinity at its C-terminal domain. The interaction of AS with tau has been probed by NMR,^51^ showing that the region that engages in tau interactions is the negatively charged C-terminal of AS, involving residues 101–140. The MTBD of tau is rich in Lys and Arg residues, and hence, it is positively charged at neutral pH, and it is likely to engage into electrostatic interactions with the negatively charged C-terminal domain of AS. AS binds several metal ions at its C-terminal domain, including Cu^2+^, Zn^2+^, Fe^2+^ and Mn^2+^, via its DPDNEA motif encompassing residues 119–125.^52^ Hence, these transition metal ions might compete, participate or interfere with AS-tau interactions. Specifically, for copper and zinc ions, given their coordination sphere in their tau-bound form at the MTBD region (Figure 2), it is tempting to propose that the DPDNEA motif from AS could provide an appealing ligand to yield a ternary AS-metal-tau complex that would be favored by the electrostatic interaction between the two proteins; although the formation of such ternary complex has not been probed. Conversely, metal binding to AS at its C-terminal domain might reduce the electrostatic interaction with the MTBD region of tau and reduce AS-tau interactions. Clearly, the role of metal binding in AS-tau interactions deserves further study, and becomes relevant in both, physiological and pathological context, since both proteins bind to microtubules and tubulin binding modulates the functional conversion of AS-tau condensates into microtubules, preventing their aggregation.^53^

Another metal-binding protein that interacts with the MTBD of tau is the cellular prion protein (PrP^C^). Increasing evidence indicates that PrP^C^ acts as a key mediator of tau toxicity through its ability to bind extracellular tau assemblies.^54^ Indeed, PrP^C^ interacts with multiple recombinant human tau fibrillar species containing the MTBD; these include full-length Tau441, as well as truncated aggregation-prone fragments such as the K18 construct,^55^ the extended 244–378 fragment, and full-length phosphorylated tau.^56^ A common feature of all these tau species is the presence of the four microtubule-binding repeats, where copper and zinc binding to tau occurs. PrP^C^ is also a metal binding protein, it can bind up to six copper ions at its disordered N-terminal region; this region can also bind zinc ions, although with lower affinity. Indeed, the suggested region of PrP^C^ engaged in the interaction of tau is its metal-binding N-terminal domain, which includes the octarepeat region.^57^ This raises the question: Could metal binding to the N-terminal sites in PrP^C^ and to the MTBD site of tau be modulating the interaction between these two metal binding proteins? And most importantly, could metal binding be enabling, mediating or modulating the uptake of fibrillar tau species by PrP^C^?

The role of metal ions in tau-PrP^C^ and tau-AS interactions gains relevance in a pathological context. AS and tau often co-deposit in Alzheimer’s disease, dementia with Lewy bodies, Parkinson’s disease dementia, and mixed AD/Lewy body cases.^58^ On the other hand, hyperphosphorylated tau has been observed surrounding or colocalizing with pathogenic or scrapie prion protein (PrP^Sc^) in plaques from patients with Gerstmann-Sträussler-Scheinker syndrome, and in sporadic and variant Creutzfeldt–Jakob Disease cases, proteinopathies caused by PrP^Sc^.^59^ Hence, tau-AS and tau-PrP interactions are of high pathological relevance, and the fact that all three proteins are metal binding proteins underscores the importance of evaluating the role of metal ions in these pathological protein–protein interactions. Unfortunately, the presence of zinc and copper in tau-AS and tau-PrP coaggregates has not been probed.

## 4 Metal Binding and Tau Aggregation

Tau is a natively unfolded and highly soluble protein with low intrinsic tendency to aggregate. However, its aggregation leads to neurodegenerative diseases characterized by deposits of tau filaments, collectively known as tauopathies.^60^ Under normal conditions, tau adopts a dynamic ensemble of transient conformations.^61^ These conformational transitions are thought to play a protective role by limiting the persistent exposure of aggregation-prone motifs, thereby reducing the intrinsic tendency of tau to self-assemble into pathological aggregates.^62^ When the electrostatics of tau are perturbed, its conformational stability is impacted, leading to the formation of oligomeric intermediates that mature into fibrillar aggregates.^63^ This can be mediated and influenced by negatively charged cofactors, such as heparin and RNA, the presence of osmolyte urea or trimethylamine N-oxide, fluctuations of the pH and the specific patterns of post-translational modifications, or other yet to be identified factors.^64,65^ Changes in the conformational state induce the opening of the protein that exposes the amyloidogenic hexapeptides PHF6 ^275^VQIINK^280^ and ^306^VQIVYK^311^ present in R2 and R3 domain respectively (Figure 1B). These motifs act as nucleation cores, driving tau aggregation via formation of a β-sheet interaction.^66,67^ Tau fibrillization follows a nucleation–elongation mechanism in which the initial nucleation step is slow and rate-limiting.^63^ This phase generates small, soluble oligomers and once the nucleus is formed, fibril elongation accelerates as monomers stack onto β-sheet-rich templates, producing paired helical filaments (PHFs) and straight filaments (SFs) in AD.^67^

Transition metal ions impact tau amyloid aggregation.^19,20,68–70^ Divalent copper and zinc ions accelerate the formation of Tau441 amyloid.^20^ Structural models of metal-bound tau (Figure 2A,C) reveal that lysine residues within the R3 repeat adopt an α-helical arrangement, positioning positively charged side chains in a geometry compatible with interactions with negatively charged cofactors, including polyanions such as heparin.^20^ This structural change may promote the spatial alignment of metal-coordinated tau monomers along polyanionic scaffolds, effectively increasing local protein concentration, and favoring intermolecular contacts. Furthermore, copper and zinc appear to differentially influence aggregate morphology. In the presence of copper, tau forms fibrils that are more cross-linked and less helical, whereas zinc promotes the formation of shorter fibrils.^20^ These distinct morphologies could arise from differences in metal coordination sites in tau, highlighting how specific metal–protein interactions may impact aggregation yielding structurally distinct tau assemblies.

Even in the absence of metal ions, tau aggregation does not result in a single uniform fibrillar species, instead it gives rise to a diverse array of structurally and morphologically distinct aggregates.^71^ These include soluble oligomers, protofibrils, PHFs, SFs, and higher order fibrillar assemblies, each exhibiting distinct biochemical properties and pathogenic potential. Advances in cryo-electron microscopy (cryo-EM) have revealed that tau filaments extracted from patients that suffer different tauopathies adopt disease specific conformations, characterized by unique β-sheet architectures, protofilament interfaces, and involvement of distinct tau sequence segments (Figure 4).^72^ This structural diversity closely correlates with tau isoform composition (3R, 4R, or 3*R*/4R) and aligns with the clinical and neuropathological signatures of individual tauopathies.^16,72^

**Figure 4:**
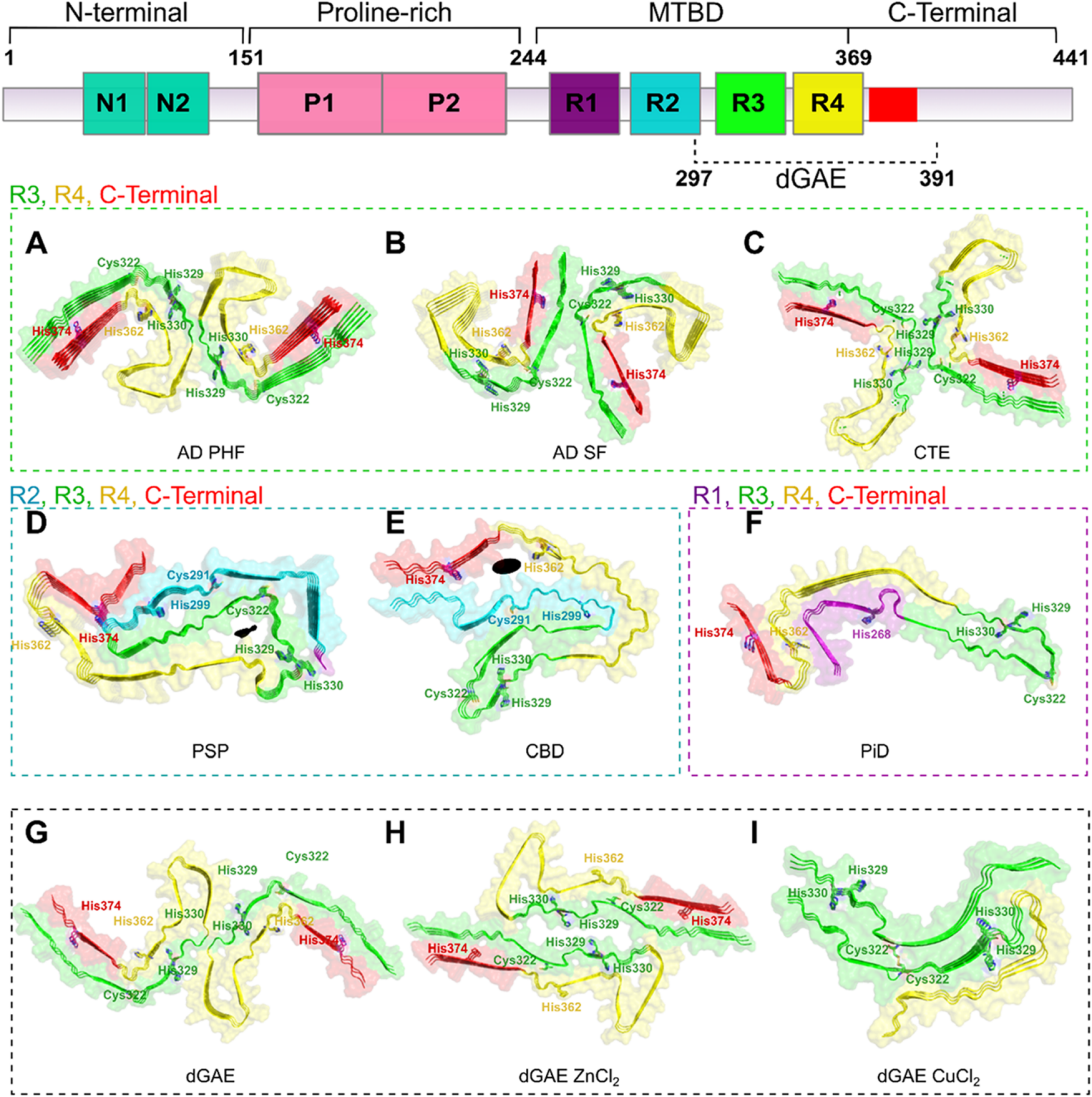
Cryo-EM structures of tau fibrils from patients with different tauopathies. Tau primary sequence, repeats are colored in a rainbow scale (R1, Purple; R2, blue; R3, green; R4, yellow; C-Terminal Red). Structure of fibrils from Alzheimer’s disease, (A) PHF, paired helical filaments (PDB: 5O3L); (B) SF, straight filaments (5O3T); (C) Chronic traumatic encephalopathy (6NWP); (D) Progressive supranuclear palsy (7P65); (E) Corticobasal degeneration (6TJO); (F) Pick’s disease (6GX5) and comparison with (G) *in vitro* obtained fibrils (7QJW) from dGAE fragment (297–391) in the presence of metal ions, (H) ZnCl_2_ (7QKL), (I) CuCl_2_ (7QKF). His and Cys residues are shown in the structures. Unresolved electron-density is shown as black holes in PSP and CBD fibrils.

The first structural description by CryoEM of tau fibrils derived from patients with AD was reported in 2017 by Fitzpatrick et al.,^72^ confirming that the core of the fibrils is composed mainly of R3 and R4 domains and includes a portion of the C-terminal region (Figure 4A,B). Cryo-EM-derived structures of tau fibrils present in other tauopathies reveal large morphological diversity, with variations in the number and type of repeats that compose the β-sheet fold of the core, and in the regions involved in the interaction between the two protofilament chains that conform the aggregates (Figure 4).^73^ For example, while the fibers found in patients with chronic traumatic encephalopathy (CTE) have a core composed of R3, R4 and a portion of the C-terminal region (Figure 4C),^74^ with a similar fold as that of AD fibrils (Figure 4A,B), the regions involved in the interaction between the two protofilaments of the fiber are different. On the other hand, tau fibrils derived from patients with progressive supranuclear palsy (PSP)^73^ or corticobasal degeneration (CBD)^75^ include the R2 domain in addition to R3 and R4 (Figure 4D,E), while fibrils from Pick′s disease (PiD)^76^ patients contain R1, instead of R2 (Figure 4F); consistent with the fact that PiD is a pathology that shows expression of the 3R isoforms only.^77^ Still, the β-sheet fold of the protofilament chains in each disease is distinct (Figure 4D–F). A common feature of the fibrils derived from PSP, CBD and PiD patients is that they are single protofilament fibrils (i.e., composed of only one chain of tau proteins), while those from AD or CTE patients are double protofilament fibrils, contributing to distinct morphologies for each disease.

The origin of the morphological diversity of tau fibrils derived from different tauopathies remains unclear. Several factors have been proposed to cause this morphological diversity in the aggregates, including differences in PTM patterns, mutations in the tau protein, differences in expression, and even the presence of transition metals. Indeed, metal ion binding to tau impacts amyloid aggregation kinetics and the final morphology of the fibrils,^20^ as discussed above. On the other hand, a Cryo-EM study using the dGAE fragment, composed by residues 297–391 of Tau441, reports distinct morphologies for the fibrils grown in the absence or presence of metal ions^78^ (Figure 4G–I). The dGAE fragment is a model used to study tau aggregation since it includes the R3 and R4 domains and the C-terminal portion that compose the core of the patient fibrils found in AD.^79^ Fibrils obtained with dGAE in the presence of copper contain an intermolecular Cys322-Cys322 disulfide bridge (Figure 4I), indicating that not only does metal binding impact the structure of the amyloid aggregates, but also its redox activity. Furthermore, although cryo-EM structures do not show the presence of metal ions, the electron density maps often display electron density that is not resolved (represented by black holes Figure 4D,E), and that it may be due to the presence of transition metal ions. In the case of PSP aggregates, unresolved electron density is observed in the center of the β-sheet structure, between Cys322 and His329 (Figure 4E), which are identified as putative ligands for both, Zn^2+^ and Cu^+^ ions in the monomeric form of tau. Hence, it would be tempting to propose that metal ion binding could impact the conformation of monomeric or oligomeric tau, resulting in diverse fibril morphology depending on metal ion availability; and ultimately, these metal ions could remain bound to the amyloid fibril, contributing to the nonresolved electron density observed by cryo-EM.

While the structural details of copper and zinc binding to monomeric tau have been elucidated^20^ there is little known about the capability of tau fibrils to bind metal ions. Given the diverse morphology described for tau fibrils (Figure 4), it is expected that copper and zinc binding sites in fibrillar tau would be equally diverse. However, it is important to note that, in all fibril structures His329 and His330, i.e., the bis-His motif in R3, end up on opposite sides of the β-sheet structure; hence, inevitably the Cu^2+^ binding site that engage this bis-His motif would be certainly perturbed upon tau fibrillation. The metal ion would likely unbind from one of the His of the bis-His motif and it might remain bound via the outer His residue. On the other hand, for Cu^+^ and Zn^2+^ ions, their binding sites in tau do not engage in coordination to both His of the bis-His motif, and hence, they might retain the same coordination upon tau fibrilization. Spectroscopic studies of tau fibrils grown in the presence of metal ions should shed light on these questions, while Cryo-EM studies of amyloid fibrils with tau should contribute to our understanding of the role of metal ions in the morphology of tau aggregates. To date, none of the Cryo-EM resolved structures of amyloid fibrils of metal binding proteins involved in neurodegenerative diseases have been modeled considering the possibility of the presence of metal ions in the structure.

## 5 Metal Binding and PTMs of Tau

Post-translational modifications play a central role in regulating both the physiological and pathological states of tau. Tau can undergo extensive phosphorylation, acetylation, ubiquitination, SUMOylation, nitration, oxidation, glycosylation, glycation, methylation, deamination, and proteolytic cleavage at numerous residues, generating a complex and highly diversity PTM landscape.^65^ Under physiological conditions, these modifications regulate tau–microtubule interactions, cytoskeletal dynamics, and protein turnover. However, pathological shifts in PTM patterns, most notably tau hyperphosphorylation reduce microtubule binding, impair clearance mechanisms, and promote tau self-association and aggregation.^79^ PTMs not just regulate tau aggregation, they also determine the morphological characteristics of the aggregates.^63^ Moreover, it is not an individual PTM that triggers aggregation, but specific combinations of PTMs that determine whether tau remains functional or transitions into neurotoxic species.^63^ Thus, dysregulation of tau PTMs represents a key molecular switch driving the conversion of a functional tau into pathogenic aggregates. In this context, it is important to note that the metal-binding sites identified on tau are located near important sites for post-translational modifications, such as phosphorylation and ubiquitination. The potential interplay between metal binding to Tau441 and its PTMs, under health and disease conditions, is discussed below.

### 5.1 Phosphorylation

Phosphorylation is one of the most studied PTM of tau, it regulates its function and it is controlled by phosphatases, such as protein phosphatase 2 (PP2A), and kinases, including glycogen synthase kinase-3 β (GSK3β) and cyclin-dependent kinase 5 (Cdk5).^23,80^ Experimental studies demonstrated that phosphorylation of tau at multiple residues (Figure 5A) reduces its affinity for microtubules, which increases microtubule dynamics and alters neurite structure.^81^ These findings suggest that controlled phosphorylation of tau can regulate cytoskeletal remodeling required for neuronal development and plasticity. Although hyperphosphorylation is the most extensively characterized and widely recognized central driver of tau dysfunction. Tau hyperphosphorylation arises from an imbalance between kinase and phosphatase activities, a dysregulation that constitutes a hallmark of multiple tauopathies, including Alzheimer’s disease. Specific phosphorylation events at residues S202, T205, T212, S214, T231, S262, S320, and S324 have been consistently associated with pathological tau states, while modifications at T217 and T231 show strong correlations with cognitive decline and disease progression.^82^

**Figure 5:**
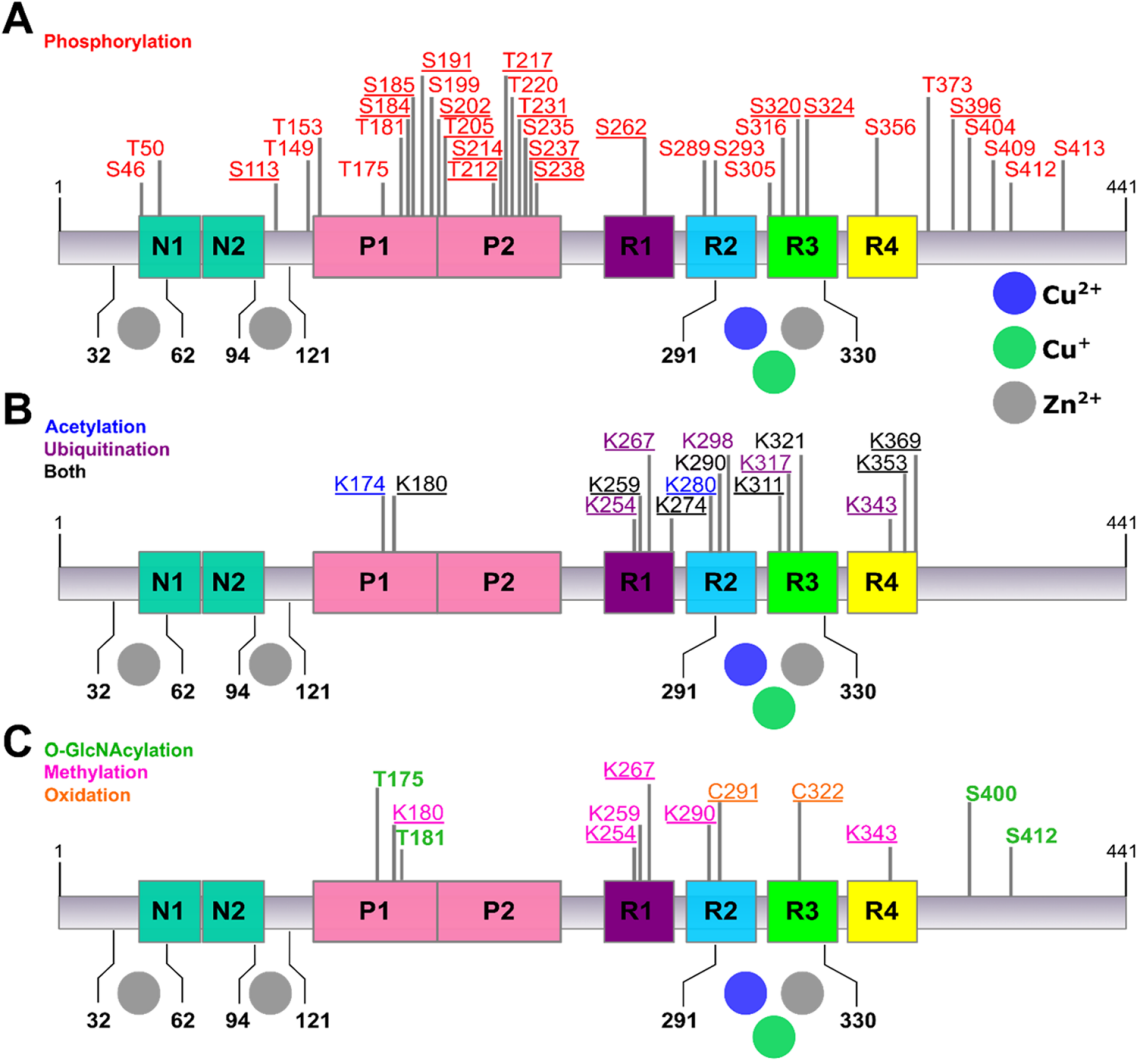
Metal Binding sites and post-translational modifications of Tau441. Cu^2+^, Cu^+^ and Zn^2+^ coordination sites in Tau441 are identified by blue, green and gray circles, respectively. The location of Tau441 phosphorylation, acetylation, ubiquitination, o-GlcNAcylation, methylation and Cys oxidation sites are shown above: (A) phosphorylation occurs at different Thr and Ser residues (marked in red), (B) acetylation occurs at the Lys residues (marked in blue) and ubiquitination at certain Lys residues (marked in purple). Both ubiquitination and acetylation occur in some Lys residues (marked in black). (C) Methylation also occurs in some Lys residues (marked in pink). Cys undergo oxidation (marked in orange) and o-GlcNAcylation occurs in some Ser/Thr residues (marked in green). Residues with pathological-associated PTMs are underlined. Figure Adapted from ref 20 with permission of Royal Society of Chemistry.

Tau phosphorylation may impact metal binding, in fact, a study with Tau441 incubated *in vitro* with GSK3β shows that phosphorylation of the protein decreases its affinity for zinc, while copper binding is abrogated.^70^ Tau phosphorylation by GSK3β occurs at Ser residues in the R2 and R3 domains,^83^ namely Ser289, Ser305 and Ser324, which are right in between the amino acids that have been identified as copper and zinc anchoring residues (Figure 5A). Hence, it is likely that phosphorylation at such Ser residues would impact significantly both, copper and zinc binding to the MTBD region, a hypothesis that would need to be probed by spectroscopic studies of metal binding to phosphorylated Tau441. Furthermore, Ser46 and Thr50 residues are also phosphorylated by GSK3β,^83^ which could impact zinc-binding to the first N-terminal site in tau, leaving only the second site in this region available for zinc binding (Figure 5A).

Phosphorylation occurs at several other sites that are not in proximity to tau’s metal binding sites (Figure 5A); these phosphorylation events may influence metal interactions only indirectly, through conformational effects or by altering tau–microtubule association. Furthermore, zinc and copper may play a role in regulating the phosphorylation of tau, since *in vitro* and *in vivo* studies show that copper and zinc cause an increase in the activity of GSK3β and Cdk5 kinases,^69,84^ while zinc inhibits phosphatase PP2A activity.^85^ The spatial convergence between metal binding sites and PTMs raises the possibility of a bidirectional interplay where phosphorylation modulates metal-binding affinity, while metal coordination may, in turn, influence kinase accessibility or site-specific reactivity. This hypothesis would need to be probed by spectroscopic studies of metal binding to phosphorylated Tau441.

### 5.2 Acetylation, Methylation and Ubiquitination

Acetylation, methylation and ubiquitination are PTMs that affect Lys residues and they often occur at the same site (Figure 5B,C).^23,80,86^ An example is Lys290, which undergoes physiological acetylation, methylation, and ubiquitination. Interestingly, Lys290 is adjacent to a cysteine proposed as one of the ligands Cu^+^ and Zn^2+^ ion binding.^87^ Modifications at Lys290 could therefore influence the local electrostatic environment surrounding Cys291, potentially impacting the accessibility and geometry of the metal-binding site. Although the functional consequences of these modifications remain poorly characterized, their proximity to a proposed metal-coordinating residue suggests a possible regulatory role. Under pathological conditions, aberrant acetylation, methylation and ubiquitination occur in certain Lys residues (Figure 5B,C). In fact, acetylation can block the ubiquitination of certain residues, resulting in a slow degradation of tau, which in turn leads to the accumulation and spread of pathogenic tau.^23,86^ An *ex vivo* study indicates that ubiquitination of soluble tau at residues K267, K311, and K317, together with phosphorylation of S262, are characteristic PTMs of AD patients that are distinct from those observed in other tauopathies;^88^ particularly, ubiquitination of K311 and K317 are thought to prevent tau aggregation.^89^ On the other hand, acetylation at K274 in the R1 region of MTBD is present in AD and other tauopathies,^90^ and it is known to decrease microtubule binding affinity.^91^ With respect to metal ions, a recent study using Tau441 K274Q, a mutation that mimics acetylation, shows that this modification increases zinc and copper binding affinities by 3× and 5.5× fold, respectively, as compared to WT protein.^92^ The K274Q also decreases the binding affinity of tau for DNA, suggesting potential implications for acetylated tau in the nucleus. These effects are likely electrostatic in nature, as this mutation removes a positive charge at this site, resulting in decreased electrostatic interaction with the negatively charged DNA chain, and in a decreased electrostatic repulsion with the positively charged metal ions, impacting the binding affinity of tau for these partners. While ubiquitination and acetylation occurring at Lys residues near the copper and zinc binding sites of tau (Figure 5B) could impact its metal binding properties, metal binding could also interfere with ubiquitination and acetylation of tau, potentially disturbing the regulation of its clearance mechanisms. However, further research is needed to elucidate the interplay between all these processes. Mass spectrometry studies of tau protein derived from AD patients show distinct changes in the methylation pattern of the MTBD region: methylation increases significantly at residues K254 and K311, and it is markedly reduced at K290.^93^ Lys290 also undergoes acetylation and ubiquitination, suggesting that a complex molecular interaction or competition is likely to determine the pathological tau protein methylation pattern. Furthermore, the presence of K290 and K311 in the vicinity of copper- and zinc-anchoring residues suggests that metal-tau interactions could be a critical regulator of tau′s methylation.

### 5.3 Cysteine Modifications

Although not formally considered as PTMs, cysteine modifications such as oxidation and dopamination have been observed. Dopamination of tau refers to covalent attachment of dopamine or its oxidized metabolites to cysteine residues Cys291 and Cys322, as detected in mice brain.^94^ Dopaminergic modification appears to inhibit tau aggregation,^94,95^ while enhancing its interaction with microtubules.^94^ Although copper–catalyzed dopamination has been observed in peptide fragments of tau,^96^ for physiological human Tau441 it remains to be established.

On the other hand, oxidation of tau’s cysteine residues is a redox modification that may represent a redox-sensitive regulatory hub. Tau contains two cysteines within the MTBD, Cys291 and Cys322, which can undergo reversible oxidation to form either intramolecular or intermolecular disulfide bonds.^97^ These Cys residues can interact with conserved cysteines in α- and β-tubulin, contributing to the stability and specificity of the tau–microtubule complex.^98^ Importantly, mutation of either cysteine significantly reduces Tau’s affinity for microtubules *in vivo*, indicating that they are functionally required for proper binding.^99^ Hence, the Cys291/Cys322 pair could be a redox switch that modulates tau-tubulin interactions. Since Cys291 and Cys322 also participate in Cu^+^ and Zn^2+^ binding to tau (Figure 2B,C), their oxidation would prevent metal coordination; conversely, metal binding to tau via Cys291 and Cys322 may prevent Cys oxidation under oxidative stress conditions. In any case, the involvement of these Cys residues in metal binding adds another dimension in the redox regulation of tau-tubulin interactions. A similar phenomenon has been observed for the copper chaperone Atox1, which coordinates Cu^+^ ions using two Cys residues that are influenced by the cellular redox environment.^100^ In this system, the redox state of cysteine residues modulates the copper-binding capacity of the protein; Cys oxidation prevents copper coordination and regulates copper trafficking in response to changes in intracellular redox balance. A conceptually similar mechanism could operate in tau where intracellular redox fluctuations could dynamically modulate the interplay between tau–metal and tau-tubulin interactions.

On the other hand, under oxidative conditions, cysteine oxidation becomes a critical driver of tau misfolding and aggregation by promoting the formation of disulfide-linked dimers that serve as key intermediates in fibrillization.^99^ Redox modulation of cysteine thiols directly impact tau conformational dynamics and aggregation kinetics, highlighting the importance of thiol–disulfide exchange in controlling early oligomerization events.^101^ Furthermore, disruption of cysteine residues through mutation or chemical modification markedly alters aggregation behavior and reduces the formation of pathogenic assemblies, underscoring their essential role in regulating tau misfolding and toxicity.^99^ Notably, disulfide-cross-linked tau dimers have been identified as highly potent seeding species capable of propagating tau pathology in a prion-like manner.^102^ Collectively, these findings establish cysteine oxidation not merely as a biochemical modification but as a key pathological trigger that promotes the formation of aggregation-prone tau species and facilitates disease progression.

Investigating the interplay between metal ion coordination and post-translational modifications (PTMs) in tau protein *in vivo* remains a major experimental challenge. In this context, in-cell NMR has emerged as a powerful approach, enabling the characterization of protein structure, dynamics, and interactions directly within the native intracellular environment. However, to date only two in-cell NMR studies of tau have been reported.^103,104^ In-cell NMR of tau in HEK-293-T cells reveals that the protein is primarily bound to microtubules and F-actin with MTBD residues, including PHF6.^103^ Tau in HEK cells exhibit additional signals that do not correspond to phosphorylated forms of tau, as studied *in vitro*. These signals could arise from other PTMs, such as acetylation or methylation; however, the possibility that they come from the interaction of tau with metals in the intracellular environment cannot be ruled out. In-cell NMR studies, using neurons or neuronal cell lines such as SH-SY-Y5, that probe metal binding to tau should shed light onto the interplay between metal binding, PTMs and functional tau-protein interactions.

Finally, it is interesting to note that, of all PTMs that tau protein suffers, pathological associated PTMs of tau fall more closely in the regions of metal binding sites, suggesting a stronger interplay between metal binding and PTMs associated with pathology, underscoring the importance of studying metal binding to PTM-modified forms of tau. Altogether, this discussion presents a strong case for a potential relationship between PTMs and metal binding to tau, as observed for other proteins associated with neurodegenerative diseases, such as α-synuclein, the Parkinson’s protein.^52^

## 6 Concluding Remarks

Tau is an intrinsically disordered protein that is involved in a wide range of cellular functions, beyond microtubule stabilization. It is also the main component of NFTs in Alzheimer′s disease and other tauopathies. The fact that tau emerges as a metal binding protein, capable of coordinating Cu^2+^, Cu^+^ and Zn^2+^ ions uncovers a missing mechanistic link between metal ion homeostasis and tau’s functional and pathological treats. Metal binding to tau could be remodeling tau’s conformational assemblies, modulating its interactome, impacting its posttranslational processing and influencing transitions between functional and aggregation-prone states. In this context, metal binding emerges, not only as a pathological trigger, but also as a physiological regulator that can modulate tau activity in different cellular compartments. Reclassifying tau as a metal binding protein uncovers an unprecedented bioinorganic facet of tau biology that may ultimately redefine therapeutic strategies for Alzheimer’s disease and related tauopathies.

## References

[1] Bayer T. A. (2015). Proteinopathies,
a core concept for understanding and ultimately treating degenerative
disorders?. Eur. Neuropsychopharmacol..

[2] Lee S., Choi M. C., Al Adem K., Lukman S., Kim T. Y. (2020). Aggregation
and Cellular Toxicity of Pathogenic or Non-pathogenic Proteins. Sci. Rep..

[3] Dexter D. T., Carayon A., Javoy-Agid F., Agid Y., Wells F. R., Daniel S. E., Lees A. J., Jenner P., Marsden C. D. (1991). Alterations in the levels of iron,
ferritin and other trace metals in Parkinson’s disease and
other neurodegenerative diseases affecting the basal ganglia. Brain.

[4] Posadas, Y. ; López-Guerrero, V. E. ; Arcos-López, T. ; Sayler, R. I. ; Sánchez-López, C. ; Segovia, J. ; Perez-Cruz, C. ; Quintanar, L. The Role of d-Block Metal Ions in Neurodegenerative Diseases. In Comprehensive Inorganic Chemistry III; Elsevier, 2023; pp 575–628 10.1016/B978-0-12-823144-9.00115-1.

[5] Deibel M. A., Ehmann W. D., Markesbery W. R. (1996). Copper,
iron, and zinc imbalances in severely degenerated brain regions in
Alzheimer’s disease: possible relation to oxidative stress. J. Neurol. Sci..

[6] Religa D., Strozyk D., Cherny R. A., Volitakis I., Haroutunian V., Winblad B., Naslund J., Bush A. I. (2006). Elevated
cortical zinc in Alzheimer disease. Neurology.

[7] Mezzanotte M., Stanga S. (2024). Brain Iron Dyshomeostasis and Ferroptosis in Alzheimer’s
Disease Pathophysiology: Two Faces of the Same Coin. Aging Dis..

[8] Bush A. I. (2013). The metal theory of Alzheimer’s disease. J. Alzheimer’s Dis..

[9] Lovell M. A., Robertson J. D., Teesdale W. J., Campbell J. L., Markesbery W. R. (1998). Copper,
iron and zinc in Alzheimer’s disease
senile plaques. J. Neurol. Sci..

[10] Sayre L. M., Perry G., Harris P. L., Liu Y., Schubert K. A., Smith M. A. (2000). In situ oxidative catalysis by neurofibrillary
tangles
and senile plaques in Alzheimer’s disease: a central role for
bound transition metals. J. Neurochem..

[11] Suh S. W., Jensen K. B., Jensen M. S., Silva D. S., Kesslak P. J., Danscher G., Frederickson C. J. (2000). Histochemically-reactive zinc in
amyloid plaques, angiopathy, and degenerating neurons of Alzheimer’s
diseased brains. Brain Res..

[12] Faller P., Hureau C. (2009). Bioinorganic chemistry
of copper and zinc ions coordinated
to amyloid-beta peptide. Dalton Trans..

[13] Singh S. K., Balendra V., Obaid A. A., Esposto J., Tikhonova M. A., Gautam N. K., Poeggeler B. (2022). Copper-mediated
beta-amyloid toxicity
and its chelation therapy in Alzheimer’s disease. Metallomics.

[14] Falcone E., Hureau C. (2023). Redox processes in
Cu-binding proteins: the ″in-between″ states in intrinsically
disordered peptides. Chem. Soc. Rev..

[15] Weingarten M. D., Lockwood A. H., Hwo S. Y., Kirschner M. W. (1975). A protein
factor essential for microtubule assembly. Proc.
Natl. Acad. Sci. U.S.A..

[16] Goedert M., Spillantini M. G., Jakes R., Rutherford D., Crowther R. A. (1989). Multiple isoforms
of human microtubule-associated protein
tau: sequences and localization in neurofibrillary tangles of Alzheimer’s
disease. Neuron.

[17] Soragni A., Zambelli B., Mukrasch M. D., Biernat J., Jeganathan S., Griesinger C., Ciurli S., Mandelkow E., Zweckstetter M. (2008). Structural
Characterization of Binding of Cu­(II) to
Tau Protein. Biochemistry.

[18] Roman A. Y., Devred F., Byrne D., La Rocca R., Ninkina N. N., Peyrot V., Tsvetkov P. O. (2019). Zinc Induces
Temperature-Dependent
Reversible Self-Assembly of Tau. J. Mol. Biol..

[19] Mo Z. Y., Zhu Y. Z., Zhu H. L., Fan J. B., Chen J., Liang Y. (2009). Low micromolar
zinc accelerates the fibrillization of human tau via
bridging of Cys-291 and Cys-322. J. Biol. Chem..

[20] Juárez-Romero G. U., Sun X., Gerez J. A., Auwer C. D., Landrot G., Nachtegaal M., Riek R., Luo J., Quintanar L. (2026). Structural
insights into copper and zinc binding to tau protein and the impact
of metal binding on amyloid aggregation. Chem.
Sci..

[21] Abramson J., Adler J., Dunger J., Evans R., Green T., Pritzel A., Ronneberger O., Willmore L., Ballard A. J., Bambrick J. (2024). Accurate structure prediction of biomolecular
interactions with AlphaFold 3. Nature.

[22] Kellogg E. H., Hejab N. M. A., Poepsel S., Downing K. H., DiMaio F., Nogales E. (2018). Near-atomic model of
microtubule-tau interactions. Science.

[23] Parra
Bravo C., Naguib S. A., Gan L. (2024). Cellular and pathological
functions of tau. Nat. Rev. Mol. Cell Biol..

[24] Tracy T. E., Madero-Perez J., Swaney D. L., Chang T. S., Moritz M., Konrad C., Ward M. E., Stevenson E., Huttenhain R., Kauwe G. (2022). Tau interactome maps
synaptic and mitochondrial processes associated with neurodegeneration. Cell.

[25] Ittner L. M., Ke Y. D., Gotz J. (2009). Phosphorylated
Tau interacts with
c-Jun N-terminal kinase-interacting protein 1 (JIP1) in Alzheimer
disease. J. Biol. Chem..

[26] Betters R. K., Luhmann E., Gottschalk A. C., Xu Z., Shin M. R., Ptak C. P., Fiock K. L., Radoshevich L. C., Hefti M. M. (2023). Characterization of the Tau Interactome in Human Brain
Reveals Isoform-Dependent Interaction with 14–3-3 Family Proteins. eNeuro.

[27] Gunawardana C. G., Mehrabian M., Wang X., Mueller I., Lubambo I. B., Jonkman J. E., Wang H., Schmitt-Ulms G. (2015). The Human
Tau Interactome: Binding to the Ribonucleoproteome, and Impaired Binding
of the Proline-to-Leucine Mutant at Position 301 (P301L) to Chaperones
and the Proteasome. Mol. Cell. Proteomics.

[28] Liu C., Song X., Nisbet R., Götz J. (2016). Co-immunoprecipitation
with Tau Isoform-specific Antibodies Reveals Distinct Protein Interactions
and Highlights a Putative Role for 2N Tau in Disease. J. Biol. Chem..

[29] Sultan A., Nesslany F., Violet M., Begard S., Loyens A., Talahari S., Mansuroglu Z., Marzin D., Sergeant N., Humez S. (2011). Nuclear tau, a key player in neuronal DNA protection. J. Biol. Chem..

[30] Antón-Fernández A., Valles-Saiz L., Avila J., Hernandez F. (2023). Neuronal nuclear
tau and neurodegeneration. Neuroscience.

[31] Wei Y., Qu M. H., Wang X. S., Chen L., Wang D. L., Liu Y., Hua Q., He R. Q. (2008). Binding to the minor groove of the
double-strand, tau protein prevents DNA from damage by peroxidation. PLoS One.

[32] Park C., Jung J., Hong Y., Yoo H., Yang K., Shin J., Kim M., Lim C., Jeong A., Hong S. (2026). Tau condensation on
DNA mediates microtubule attachment
suggesting a mitotic role for centromere-localized tau. Nat. Commun..

[33] Hara T., Takeda T. A., Takagishi T., Fukue K., Kambe T., Fukada T. (2017). Physiological roles
of zinc transporters: molecular
and genetic importance in zinc homeostasis. J. Physiol. Sci..

[34] Asadollahi K., Riazi G., Chadegani A. R., Rafiee S. (2018). DNA-binding mode transition
of tau in the presence of Zinc ions. J. Biomol.
Struct. Dyn..

[35] Atwa A., Alhadidy M. M., Lamp J., Combs B., Kanaan N. M. (2025). BioID2-Based
Tau Interactome Reveals Novel and Known Protein Interactions Associated
with Multiple Cellular Pathways. J. Proteome
Res..

[36] Wang C., Terrigno M., Li J., Distler T., Pandya N. J., Ebeling M., Tyanova S., Hoozemans J. J. M., Dijkstra A. A., Fuchs L. (2023). Increased G3BP2-Tau
interaction in tauopathies is a natural defense against Tau aggregation. Neuron.

[37] Ding H., Dolan P. J., Johnson G. V. (2008). Histone deacetylase
6 interacts with
the microtubule-associated protein tau. J. Neurochem..

[38] Zhang W., Zhang Y., Wang Y., Wang C., Zhang C. (2025). Advancing
histone deacetylase 6 (HDAC6) as a promising therapeutic target for
Alzheimer’s disease: from molecular insights to clinical prospects. Front. Drug Discovery.

[39] Farkas E., Bátka D., Csapó E., Buglyó P., Haase W., Sanna D. (2007). Synthesis and characterization
of
Cu^2+^, Ni^2+^ and Zn^2+^ binding capability
of some amino- and imidazole hydroxamic acids: Effects of substitution
of side chain amino-N for imidazole-N or hydroxamic-N-H for -N-CH3
on metal complexation. Polyhedron.

[40] Pooler A. M., Phillips E. C., Lau D. H., Noble W., Hanger D. P. (2013). Physiological
release of endogenous tau is stimulated by neuronal activity. EMBO Rep..

[41] Chicka M. C., Hui E., Liu H., Chapman E. R. (2008). Synaptotagmin arrests the SNARE complex
before triggering fast, efficient membrane fusion in response to Ca^2+^. Nat. Struct. Mol. Biol..

[42] Stefanoska K., Volkerling A., Bertz J., Poljak A., Ke Y. D., Ittner L. M., Ittner A. (2018). An N-terminal motif unique to primate
tau enables differential protein-protein interactions. J. Biol. Chem..

[43] Xue R., Meng H., Yin J., Xia J., Hu Z., Liu H. (2021). The Role of Calmodulin vs. Synaptotagmin
in Exocytosis. Front. Mol. Neurosci..

[44] Maret W. (2015). Analyzing
free zinc­(II) ion concentrations in cell biology with fluorescent
chelating molecules. Metallomics.

[45] Krężel A., Maret W. (2006). Zinc-buffering
capacity of a eukaryotic cell at physiological pZn. JBIC, J. Biol. Inorg. Chem..

[46] Palmiter R. D., Cole T. B., Quaife C. J., Findley S. D. (1996). ZnT-3, a putative
transporter of zinc into synaptic vesicles. Proc. Natl. Acad. Sci. U.S.A..

[47] Frederickson C. J., Suh S. W., Silva D., Frederickson C. J., Thompson R. B. (2000). Importance of zinc in the central
nervous system: the
zinc-containing neuron. J. Nutr..

[48] White Z. B., Nair S., Bredel M. (2024). The role of annexins
in central nervous system development and disease. J. Mol. Med..

[49] Gauthier-Kemper A., Weissmann C., Golovyashkina N., Sebo-Lemke Z., Drewes G., Gerke V., Heinisch J. J., Brandt R. (2011). The frontotemporal
dementia mutation R406W blocks tau’s interaction with the membrane
in an annexin A2-dependent manner. J. Cell Biol..

[50] Burre J. (2015). The Synaptic
Function of alpha-Synuclein. J. Parkinson’s
Dis..

[51] Dasari A. K. R., Kayed R., Wi S., Lim K. H. (2019). Tau Interacts with
the C-Terminal Region of alpha-Synuclein, Promoting Formation of Toxic
Aggregates with Distinct Molecular Conformations. Biochemistry.

[52] González N., Arcos-Lopez T., Konig A., Quintanar L., Marquez M. M., Outeiro T. F., Fernandez C. O. (2019). Effects
of alpha-synuclein post-translational modifications on metal binding. J. Neurochem..

[53] Padilla-Godinez F. J., Vazquez-Garcia E. R., Trujillo-Villagran M. I., Soto-Rojas L. O., Palomero-Rivero M., Hernandez-Gonzalez O., Perez-Eugenio F., Collazo-Navarrete O., Arias-Carrion O., Guerra-Crespo M. (2025). alpha-synuclein
and tau: interactions, cross-seeding, and the redefinition of synucleinopathies
as complex proteinopathies. Front. Neurosci..

[54] Gavin R., Del Rio J. A. (2025). Exploring the Biological
Connection Between Tau and
PrP­(C) in Neuronal Cells: GSK3beta as a Possible Key Player. Mol. Neurobiol..

[55] De
Cecco E., Celauro L., Vanni S., Grandolfo M., Bistaffa E., Moda F., Aguzzi A., Legname G. (2020). The uptake
of tau amyloid fibrils is facilitated by the cellular prion protein
and hampers prion propagation in cultured cells. J. Neurochem..

[56] Celauro L., Burato A., Zattoni M., De Cecco E., Fantuz M., Cazzaniga F. A., Bistaffa E., Moda F., Legname G. (2023). Different
tau fibril types reduce prion level in chronically and de novo infected
cells. J. Biol. Chem..

[57] Wang X. F., Dong C. F., Zhang J., Wan Y. Z., Li F., Huang Y. X., Han L., Shan B., Gao C., Han J., Dong X. P. (2008). Human tau
protein forms complex with PrP and some GSS-
and fCJD-related PrP mutants possess stronger binding activities with
tau in vitro. Mol. Cell. Biochem..

[58] Galloway P. G., Bergeron C., Perry G. (1989). The presence
of tau distinguishes Lewy bodies of diffuse Lewy body disease from
those of idiopathic Parkinson disease. Neurosci.
Lett..

[59] Ghetti B., Tagliavini F., Masters C. L., Beyreuther K., Giaccone G., Verga L., Farlow M. R., Conneally P. M., Dlouhy S. R., Azzarelli B., Bugiani O. (1989). Gerstmann-Straussler-Scheinker disease. II. Neurofibrillary
tangles and plaques with PrP-amyloid coexist in an affected family. Neurology.

[60] Kovacs G. G., Ghetti B., Goedert M. (2022). Classification
of diseases with accumulation
of Tau protein. Neuropathol. Appl. Neurobiol..

[61] Skrabana R., Skrabanova-Khuebachova M., Kontsek P., Novak M. (2006). Alzheimer’s-disease-associated
conformation of intrinsically disordered tau protein studied by intrinsically
disordered protein liquid-phase competitive enzyme-linked immunosorbent
assay. Anal. Biochem..

[62] Chen D., Drombosky K. W., Hou Z., Sari L., Kashmer O. M., Ryder B. D., Perez V. A., Woodard D. R., Lin M. M., Diamond M. I., Joachimiak L. A. (2019). Tau local
structure shields an amyloid-forming
motif and controls aggregation propensity. Nat.
Commun..

[63] Limorenko G., Lashuel H. A. (2022). Revisiting the grammar of Tau aggregation
and pathology
formation: how new insights from brain pathology are shaping how we
study and target Tauopathies. Chem. Soc. Rev..

[64] Goedert M., Jakes R., Spillantini M. G., Hasegawa M., Smith M. J., Crowther R. A. (1996). Assembly of microtubule-associated
protein tau into
Alzheimer-like filaments induced by sulphated glycosaminoglycans. Nature.

[65] Jebarupa B., Muralidharan M., Arun A., Mandal A. K., Mitra G. (2018). Conformational
heterogeneity of tau: Implication on intrinsic disorder, acid stability
and fibrillation in Alzheimer’s disease. Biophys. Chem..

[66] von
Bergen M., Friedhoff P., Biernat J., Heberle J., Mandelkow E. M., Mandelkow E. (2000). Assembly of tau protein into Alzheimer
paired helical filaments depends on a local sequence motif ((306)­VQIVYK(311))
forming beta structure. Proc. Natl. Acad. Sci.
U.S.A..

[67] von
Bergen M., Barghorn S., Li L., Marx A., Biernat J., Mandelkow E. M., Mandelkow E. (2001). Mutations
of tau protein in frontotemporal dementia promote aggregation of paired
helical filaments by enhancing local beta-structure. J. Biol. Chem..

[68] Ahmadi S., Zhu S., Sharma R., Wu B., Soong R., Majumdar R. D., Wilson D. J., Simpson A. J., Kraatz H. B. (2019). Aggregation of Microtubule Binding Repeats of Tau Protein
is Promoted by Cu(2). ACS Omega.

[69] Kitazawa M., Cheng D., Laferla F. M. (2009). Chronic
copper exposure exacerbates
both amyloid and tau pathology and selectively dysregulates cdk5 in
a mouse model of AD. J. Neurochem..

[70] Ahmadi S., Zhu S., Sharma R., Wilson D. J., Kraatz H. B. (2019). Interaction of metal
ions with tau protein. The case for a metal-mediated tau aggregation. J. Inorg. Biochem..

[71] Van
Alstyne M., Pratt J., Parker R. (2025). Diverse influences
on tau aggregation and implications for disease progression. Genes Dev..

[72] Fitzpatrick A. W. P., Falcon B., He S., Murzin A. G., Murshudov G., Garringer H. J., Crowther R. A., Ghetti B., Goedert M., Scheres S. H. W. (2017). Cryo-EM
structures of tau filaments from Alzheimer’s
disease. Nature.

[73] Shi Y., Zhang W., Yang Y., Murzin A. G., Falcon B., Kotecha A., van Beers M., Tarutani A., Kametani F., Garringer H. J. (2021). Structure-based classification of tauopathies. Nature.

[74] Falcon B., Zivanov J., Zhang W., Murzin A. G., Garringer H. J., Vidal R., Crowther R. A., Newell K. L., Ghetti B., Goedert M., Scheres S. H. W. (2019). Novel tau filament
fold in chronic
traumatic encephalopathy encloses hydrophobic molecules. Nature.

[75] Zhang W., Tarutani A., Newell K. L., Murzin A. G., Matsubara T., Falcon B., Vidal R., Garringer H. J., Shi Y., Ikeuchi T. (2020). Novel
tau filament fold in corticobasal
degeneration. Nature.

[76] Falcon B., Zhang W., Murzin A. G., Murshudov G., Garringer H. J., Vidal R., Crowther R. A., Ghetti B., Scheres S. H. W., Goedert M. (2018). Structures of filaments
from Pick’s
disease reveal a novel tau protein fold. Nature.

[77] Tamvaka N., Soto-Beasley A. I., Gavrielatos M., Heckman M. G., Ren Y., Udine E., Quicksall Z. S., Liskey D., Castanedes-Casey M., Wszolek Z. K. (2025). Characterizing the expression profile of 3R
tau pathology in Pick’s disease. Sci.
Adv..

[78] Lovestam S., Koh F. A., van Knippenberg B., Kotecha A., Murzin A. G., Goedert M., Scheres S. H. W. (2022). Assembly of recombinant tau into
filaments identical to those of Alzheimer’s disease and chronic
traumatic encephalopathy. eLife.

[79] Bramblett G. T., Goedert M., Jakes R., Merrick S. E., Trojanowski J. Q., Lee V. M. (1993). Abnormal tau phosphorylation at Ser396 in Alzheimer’s
disease recapitulates development and contributes to reduced microtubule
binding. Neuron.

[80] Wang Y., Mandelkow E. (2016). Tau in physiology
and pathology. Nat. Rev. Neurosci..

[81] Lu S., Qiu S., Guan Y., Zhang A., Zhao Q. (2026). Tau phosphorylation
homeostasis: Mechanisms, targets, and therapeutic implications in
Alzheimer’s disease. Ageing Res. Rev..

[82] Barthélemy N.
R., Bateman R. J., Hirtz C., Marin P., Becher F., Sato C., Gabelle A., Lehmann S. (2020). Cerebrospinal fluid phospho-tau T217
outperforms T181 as a biomarker for the differential diagnosis of
Alzheimer’s disease and PET amyloid-positive patient identification. Alzheimer’s Res. Ther..

[83] Hanger D. P., Noble W. (2011). Functional implications
of glycogen synthase kinase-3-mediated tau
phosphorylation. Int. J. Alzheimer’s
Dis..

[84] Voss K., Harris C., Ralle M., Duffy M., Murchison C., Quinn J. F. (2014). Modulation of tau
phosphorylation by environmental copper. Transl.
Neurodegener..

[85] Xiong Y., Jing X. P., Zhou X. W., Wang X. L., Yang Y., Sun X. Y., Qiu M., Cao F. Y., Lu Y. M., Liu R., Wang J. Z. (2013). Zinc induces
protein phosphatase 2A inactivation and tau hyperphosphorylation through
Src dependent PP2A (tyrosine 307) phosphorylation. Neurobiol. Aging.

[86] Kontaxi C., Piccardo P., Gill A. C. (2017). Lysine-Directed
Post-translational
Modifications of Tau Protein in Alzheimer’s Disease and Related
Tauopathies. Front. Mol. Biosci..

[87] Cohen T. J., Guo J. L., Hurtado D. E., Kwong L. K., Mills I. P., Trojanowski J. Q., Lee V. M. (2011). The acetylation of tau inhibits its function and promotes
pathological tau aggregation. Nat. Commun..

[88] Zola N. K. N., Balty C., Ruys S. P. D., Vanparys A. A. T., Huyghe N. D. G., Herinckx G., Johanns M., Boyer E., Kienlen-Campard P., Rider M. H. (2023). Specific post-translational modifications of
soluble tau protein distinguishes Alzheimer’s disease and primary
tauopathies. Nat. Commun..

[89] Wang P., Joberty G., Buist A., Vanoosthuyse A., Stancu I. C., Vasconcelos B., Pierrot N., Faelth-Savitski M., Kienlen-Campard P., Octave J. N. (2017). Tau interactome mapping based identification
of Otub1 as Tau deubiquitinase involved in accumulation of pathological
Tau forms in vitro and in vivo. Acta Neuropathol..

[90] Tracy T. E., Sohn P. D., Minami S. S., Wang C., Min S. W., Li Y., Zhou Y., Le D., Lo I., Ponnusamy R. (2016). Acetylated Tau Obstructs KIBRA-Mediated Signaling in Synaptic Plasticity
and Promotes Tauopathy-Related Memory Loss. Neuron.

[91] Rane J. S., Kumari A., Panda D. (2019). An acetylation mimicking
mutation,
K274Q, in tau imparts neurotoxicity by enhancing tau aggregation and
inhibiting tubulin polymerization. Biochem.
J..

[92] Rane J. S., Kumari A., Panda D. (2020). The Acetyl Mimicking Mutation, K274Q
in Tau, Enhances the Metal Binding Affinity of Tau and Reduces the
Ability of Tau to Protect DNA. ACS Chem. Neurosci..

[93] Balmik A. A., Chinnathambi S. (2021). Methylation as a key regulator of Tau aggregation and
neuronal health in Alzheimer’s disease. Cell Commun. Signaling.

[94] Wang Q., Liu Z., Wang Y., Liu Y., Chen Y., Zhang S., Zeng W., Li D., Yang F., He Z. (2025). Quantitative chemoproteomics
reveals dopamine’s protective
modification of Tau. Nat. Chem. Biol..

[95] Liu Z., Li X., Wang Q., Liu K., Zeng W., Li D., Zhao K., Ma Y., Long H., Zhang S. (2026). Dopamine-Induced Tau Modification Prevents Pathological Phosphorylation
and Generates a Distinct Fibril Polymorph. J.
Am. Chem. Soc..

[96] Bacchella C., Gentili S., Mozzi S. I., Monzani E., Casella L., Tegoni M., Dell’Acqua S. (2022). Role of the
Cysteine in R3 Tau Peptide
in Copper Binding and Reactivity. Int. J. Mol.
Sci..

[97] Hatters D. M. (2021). Flipping
the switch: How cysteine oxidation directs tau amyloid conformations. J. Biol. Chem..

[98] Martinho M., Allegro D., Huvent I., Chabaud C., Etienne E., Kovacic H., Guigliarelli B., Peyrot V., Landrieu I., Belle V., Barbier P. (2018). Two Tau binding
sites on tubulin
revealed by thiol-disulfide exchanges. Sci.
Rep..

[99] Prifti E., Tsakiri E. N., Vourkou E., Stamatakis G., Samiotaki M., Papanikolopoulou K. (2021). The Two Cysteines of Tau Protein
Are Functionally Distinct and Contribute Differentially to Its Pathogenicity
in Vivo. J. Neurosci..

[100] Hatori Y., Lutsenko S. (2016). The Role of Copper
Chaperone Atox1
in Coupling Redox Homeostasis to Intracellular Copper Distribution. Antioxidants.

[101] Furukawa Y., Kaneko K., Nukina N. (2011). Tau protein assembles
into isoform- and disulfide-dependent polymorphic fibrils with distinct
structural properties. J. Biol. Chem..

[102] Kim D., Lim S., Haque M. M., Ryoo N., Hong H. S., Rhim H., Lee D. E., Chang Y. T., Lee J. S., Cheong E. (2015). Identification of disulfide cross-linked tau dimer
responsible for tau propagation. Sci. Rep..

[103] Zhang S., Wang C., Lu J., Ma X., Liu Z., Li D., Liu Z., Liu C. (2019). In-Cell NMR
Study of
Tau and MARK2 Phosphorylated Tau. Int. J. Mol.
Sci..

[104] Cherot H., Pred’homme T., Thai R., Theodoro F., Castelli F., Theillet F. X. (2025). In-Cell Residue-Resolved NMR of Micromolar
alpha-Synuclein and Tau at 310 K. J. Am. Chem.
Soc..

